# Comparison of thiafentanil-medetomidine to etorphine-medetomidine immobilisation of impalas (*Aepyceros melampus*)

**DOI:** 10.4102/jsava.v88i0.1520

**Published:** 2017-08-04

**Authors:** Gareth E. Zeiler, Leith C.R. Meyer

**Affiliations:** 1 Department of Paraclinical Sciences, University of Pretoria, South Africa

## Abstract

Impalas (*Aepyceros melampus*) are increasingly valuable in the South African wildlife industry, and there is a greater need to chemically immobilise them, ideally with minimal risk. This study aimed to compare the times to recumbency and physiological effects of thiafentanil-medetomidine versus etorphine-medetomidine immobilisation. A combination of thiafentanil (2 mg) + medetomidine (2.2 mg) and etorphine (2 mg) + medetomidine (2.2 mg) was administered (to nine impalas; crossover design) via a dart. After darting, a stopwatch was started to record times to recumbency (time from darting until recumbent without attempts to stand). If apnoea was present, the impalas received one or more boluses of butorphanol (1:1 potent opioid dose). Data collection included arterial blood gas analysis and the number of butorphanol boluses. Two-sample *t*-tests were used to compare differences between combinations. The time to recumbency for thiafentanil-medetomidine was 12.2 (± 6.8) min and no different from 14.5 (± 5.2) min for etorphine-medetomidine (*p* = 0.426). The thiafentanil-medetomidine combination required more butorphanol boluses (median: 2; interquartile range: 2–3) compared to etorphine-medetomidine (median: 0; interquartile range: 0–1) (*p* = 0.001). Despite butorphanol treatment and resolution of apnoea, all impalas suffered hypoxaemia (PaO_2_ ± 44.0 mmHg). Thiafentanil-medetomidine did not immobilise impalas more rapidly than etorphine-medetomidine, and resulted in more apnoea that required rescue butorphanol boluses. Marked hypoxaemia resulted from both combinations, mainly because of right-to-left intrapulmonary shunting and not because of hypoventilation. Butorphanol and oxygen supplementation should be considered as essential rescue interventions for all impalas immobilised with these potent opioid combinations.

## Introduction

Impalas (*Aepyceros melampus*) have been commonly used as a research animal model for antelope immobilisation in recent years (Meyer et al. 2008; Zeiler et al. 2015). This increased interest is because of impalas and other antelopes being increasingly valuable in the South African wildlife industry; thus, they are commonly immobilised for translocation, and owners are more prepared to consent to invasive surgical interventions and other veterinary treatments in injured animals. To achieve these goals, antelopes need to be immobilised safely. Because of an inherent flight response and nervous disposition, impalas make an excellent animal model to study immobilisation protocols in antelopes (Knox, Hattingh & Raath 1990).

Thiafentanil and etorphine are potent opioids commonly used to immobilise antelope because of their rapid, predictable and reversible effects (Pienaar et al. 1966). Thiafentanil is a fentanyl analogue (of the 4-anilidopiperidine opioid class) and, like fentanyl, has an exclusive affinity for the mu-opioid receptor (Vardanyan & Hruby 2014). It is the next generation of opioid immobilisation drugs, which is claimed to induce more rapid immobilisation and shorter duration of action compared to etorphine (Lance & Kenny 2012). Also, claims that thiafentanil produces less respiratory and cardiac depression, when compared with other potent opioids such as fentanyl, carfentanil and etorphine, exist (Lance & Kenny 2012). Furthermore, thiafentanil is claimed to be the opioid drug of choice in impala immobilisation (Burroughs et al. 2012). Whereas etorphine is a semi-synthetic derivative of the opioid alkaloid thebaine (belonging to the phenanthrene chemical opioid class), which is a non-specific agonist at the mu-, kappa- and delta opioid receptors (Blane et al. 1967; Yaksh & Wallace 2011), medetomidine is a specific alpha_2_-adrenoceptor agonist used as a synergist with potent opioids in immobilisation drug combinations. It is used for its reliable sedative, good muscle relaxation and analgesic properties (Kästner 2006).

This study aimed to evaluate and compare the times to recumbency and physiological effects of thiafentanil-medetomidine versus etorphine-medetomidine immobilisation in impalas.

## Materials and methods

The study reported here was part of a larger collaborative study evaluating different total intravenous anaesthetic (TIVA) maintenance protocols in immobilised impalas, reported elsewhere (Buck et al. 2017; Gerlach et al. 2017; Zeiler et al. 2015). The data reported in this study compare two immobilisation protocols used in the collaborative study, whereas the TIVA studies focus on reporting the pharmacodynamic characteristics of the maintenance protocols alone, without comparisons between the two immobilisation protocols described herein.

### Animals and study area

Nine adult female impalas were first habituated (6 weeks prior to drug trials) to captivity in a boma (outdoor antelope holding facility) and then enrolled into the prospective crossover study. The boma was made up of two adjoining partitions: a smaller feeding area (150 m^2^) and a larger home area (300 m^2^). The partitions were divided by an internal shade cloth-covered wire fence (same construct as the boma perimeter fencing) with inter-leading swing-gates at both ends of the partition wall (Zeiler & Meyer in press). Thirty minutes prior to the chemical capture, the impalas were confined to the feeding area and allowed to rest.

### Procedures

The impalas were immobilised with thiafentanil-medetomidine and then 4 weeks later with etorphine-medetomidine. The doses of the drugs used in the immobilisation were calculated for a 40-kg impala, as follows:

- Thiafentanil-medetomidine: thiafentanil (2.0 mg; Thianil 10 mg/mL; Wildlife Pharmaceuticals; Karino, South Africa) + medetomidine (2.2 mg; Medetomidine 10 mg/mL; Kyron Prescriptions; Benrose, South Africa).
- Etorphine-medetomidine: etorphine (2.0 mg; Captivon 9.8 mg/mL; Wildlife Pharmaceuticals) + medetomidine (2.2 mg).

The drugs were administered into suitable muscles of the pelvic girdle using a 3-mL dart (Dan-Inject 3 mL, 25 mm plain needle; S300 Syringe Dart; Dan-inject International SA; Skukuza, South Africa) projected over a 10 m – 15 m distance from a carbon dioxide-powered dart rifle (Model JM; Dan-inject International SA). The drugs were added to the dart and then the drug chamber was filled to 3 mL by adding sterile water for injection (Kyron Laboratories Ltd., Benrose, South Africa). Once the dart was placed and discharged, a stopwatch was started to record the time of events. Time to recumbency was defined as the time from dart placement until the impalas became recumbent (sternal or lateral) without attempts to stand. Once recumbent, the remaining herd was shepherded into and confined to the home area of the boma. The immobilised impala was blindfolded and cotton wool swabs were placed into the ear canals to minimise external visual and auditory stimulation. One of the cephalic veins was aseptically cannulated using an over-the-needle catheter (20 Gauge; Jelco; Smiths Medical; Lancashire, UK). A basic field clinical examination (temperature, heart and respiratory rates) was conducted. The impala was loaded onto the back of a pickup truck and driven to the procedure room 650 m away. During this period, the impala’s respiratory rate and effort were monitored, and if apnoea (no visible breath attempt for 60 s) or dyspnoea or cyanosis was detected, then a single bolus of butorphanol (2 mg; Butorphanol 10 mg/mL; V-Tech Pharmacy; Pretoria, South Africa) was administered intravenously. Additional butorphanol boluses were administered if the initial response (improved respiratory rate without signs of arousal) was unsatisfactory. The total number of butorphanol boluses required to achieve the intended response was recorded.

Once in the procedure room, the temperature, heart and respiratory rates were measured again. The auricular artery was aseptically cannulated using an over-the-needle catheter (22 Gauge; Jelco). Arterial blood was collected anaerobically in a pre-heparinised syringe and analysed immediately using a patient-side gas analyser (EPOC Reader Blood Analysis and EPOC BGEM smart cards; Epocal; Kyron Laboratories). Information from the blood gas analysis important to this study included the arterial oxygen (PaO_2_) and carbon dioxide (PaCO_2_) tensions, lactate concentration and barometric pressure. The arterial blood pressure was measured using an electronic strain gauge (BD DTX; Bacton and Dickson Medical; NY, USA) zeroed to atmospheric air pressure at the level of the right atrium) coupled to a multiparameter monitoring machine (Datex-Ohmeda S/5 Anesthesia Monitor; GE Healthcare; Finland). The trachea of the impala was intubated with a cuffed endotracheal tube (size 8), and the animal was allowed to breathe spontaneously. A pitot-tube and side-stream respiratory gas analyser (200 mL/min sampling rate) was coupled to the end of the endotracheal tube and connected to the multiparameter monitoring machine to measure minute volume and end-tidal carbon dioxide pressure (P_ET_CO_2_). A venous blood sample was collected from the lateral saphenous vein and stored in a serum tube (BD Vacutainer tube; BD Diagnostics; New Jersey, USA). The time from darting until completion of all data collection for the impala was recorded (time to data collection completion). The venous blood samples were allowed to clot (60 min; room temperature 18 °C – 23 °C) prior to centrifugation (5000G for 10 min) to separate the cellular and fluid components of the blood. The serum was carefully pipetted into cryovials and stored in a -80 °C freezer until serum cortisol concentration determination. Total serum cortisol concentration was determined (Immulite 1000; Siemens; Isando, South Africa) using a chemiluminescent enzyme immunoassay.

The impala was kept under general anaesthesia for 120 min. After the general anaesthesia, the impala were returned to the feeding area of the boma for an uneventful recovery (Buck et al. 2017; Zeiler et al. 2015).

### Data analysis

The two data sets (obtained in the boma and procedure room) for temperature, heart and respiratory rates were averaged for each impala prior to analysis. The following equation was used to calculate the alveolar oxygen tension: PAO_2_ = FiO_2_(Patm – PH_2_O) – PaCO_2_/RQ; where FiO_2_ is the fraction of inspired oxygen (21% for room air); Patm is the barometric pressure (measured during the study); PH_2_O is the water vapour tension in the alveoli (47 mmHg at 37 °C, uncorrected for body temperature); and RQ is the respiratory quotient (1.0 for ruminants). The alveolar-to-arterial oxygen tension [P(A-a)O_2_] gradient was calculated by subtracting the measured arterial oxygen tension (PaO_2_) from the calculated alveolar oxygen tension (PAO_2_). The arterial-to-end-tidal carbon dioxide tension (PaCO_2_-P_ET_CO_2_) gradient was calculated by subtracting the measured end-tidal carbon dioxide tension (P_ET_CO_2_) from the measured arterial carbon dioxide tension (PaCO_2_).

Data were assessed for normality by evaluating descriptive statistics, plotting of histograms and performing the Anderson–Darling test for normality. Parametric quantitative data [heart rate, respiratory rate, mean arterial blood pressure, cortisol concentration, temperature, PaO_2_, PaCO_2_, PaCO_2_-P_ET_CO_2_ and P(A-a)O_2_ gradients] were compared between groups using two-sample *t*-tests. Non-parametric data (number of butorphanol boluses, lactate concentration) were compared using Moods Median Test. Data were reported as mean (±s.d.: standard deviation) unless otherwise stated. Scatter plots and general linear regression were used to compare cortisol concentrations to times to recumbency within and between the two drug protocols. Correlations between variables of interest (times to recumbency, cortisol concentrations) were determined using the Pearson correlation test. Data were analysed using commercially available software (MiniTab 17.1.0; MiniTab Incorporated; State College, Pennsylvania, USA) and results interpreted at the 5% level of significance.

## Results

The study was conducted at an altitude of 1252 m above sea level, where the average barometric pressure was 665 mmHg (88.6 kPa). The weight of the impalas averaged 38.6 (± 4.3) kg and 40.4 (± 4.2) kg during the thiafentanil-medetomidine and etorphine-medetomidine data collection sessions, respectively (*p* = 0.371). Thus the mean (range) total dose for thiafentanil and medetomidine was 0.052 (0.047–0.058) mg/kg and 0.057 (0.051–0.064) mg/kg, respectively; and for etorphine and medetomidine was 0.050 (0.045–0.055) mg/kg and 0.054 (0.49–0.061) mg/kg, respectively. All impalas were successfully immobilised with both drug combinations.

**FIGURE 1:**
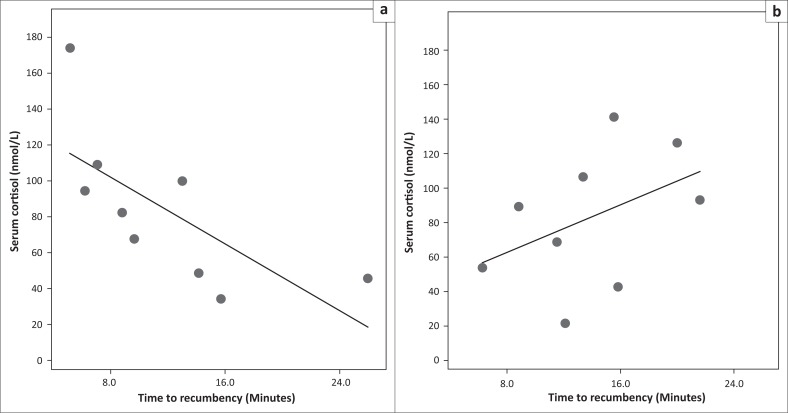
Scatter plots with general linear modelling demonstrating the significant difference (*p* = 0.023) of cortisol concentration (nmol/L) of impala (*Aepyceros melampus*) versus the time to recumbency (minutes) between (a) thiafentanil-medetomidine and (b) etorphine-medetomidine.

The overall time to recumbency for thiafentanil-medetomidine was 12.2 (± 6.8) min, which was no different from 14.5 (± 5.2) min for the etorphine-medetomidine combination (*p* = 0.426). However, the association between cortisol concentrations and times to recumbency differed significantly between the immobilisation combinations (*p* = 0.023; slopes and intercepts on linear regression graphs; Figure 1). The cortisol concentrations versus times to recumbency demonstrated a strong negative correlation (*p* = 0.036; *r* = -0.699) when the thiafentanil-medetomidine drug combination was used compared to no correlation (*p* = 0.238; *r* = 0.438) for the etorphine-medetomidine combination. Nonetheless, the overall cortisol concentrations did not differ between the combinations (Table 1). The overall times to data collection completion were 35.5 (± 8.4) and 32.2 (± 8.3) min for thiafentantil-medetomidine and etorphine-medetomidine, respectively (*p* = 0.424).

**TABLE 1:** Physiological variables obtained from impalas (*Aepyceros melampus*) immobilised with thiafentanil-medetomidine and etorphine-medetomidine combinations.

Parameter	Unit	Thiafentanil-medetomidine			Etorphine-medetomidine			*p*-value (*t*-tests)
		Mean	± s.d.	Range	Mean	± s.d.	Range	
**Clinical examination measurements**								
Temperature	°C	38.9	±1.1	37.6–40.8	39.3	±0.7	38.2–40.6	0.341
Heart Rate	Beats/min	122	±40	71–197	79	±37	45–150	0.032
Respiratory Rate	Breaths/min	9	±5	2–15	9	±2	2–13	0.818
Minute volume	L/min	8.9	±4.7	2.4–14.3	8.0	±3.2	3.5–14.0	0.659
MAP	mmHg	126	±14	108–152	117	±18	94–133	0.226
**Clinical pathology measurements and calculations**								
PaO_2_	mmHg	47	±8	38–62	41	±12	24–55	0.193
	kPa	6.3	±1.1	5.1–8.3	5.5	±1.6	3.2–7.3	
PaCO_2_	mmHg	51	±9	38–68	51	±7	45–63	0.952
	kPa	6.8	±1.2	5.1–9.1	6.8	±0.9	6.0–8.4	
P_ET_CO_2_	mmHg	41	±7	29–52	51	±7	41–64	0.019
	kPa	5.5	±0.9	3.9–6.9	6.8	±0.9	5.5–8.5	
PaCO_2_-P_ET_CO_2_	mmHg	10	±9	(-4)–23	0	±8	(-11)–12	0.028
P(A-a)O_2_	mmHg	31	±7	19–40	38	±8	29–48	0.078
Lactate	mmol/L	2.2^a^	(1.8–4.9)^a^	1.4–5.4	2.2^a^	(1.9–4.2)^a^	1.2–5.3	0.637^b^
Cortisol	nmol/L	84.5	±41.9	35.6–173.0	80.0	±37.7	21.8–136.0	0.817

s.d., standard deviation; °C, degree Celsius; min, minute; MAP, mean arterial blood pressure; mmHg, millimetres mercury; kPa, kilopascals; PaO_2_, arterial oxygen tension; PaCO_2_, arterial carbon dioxide tension; P_ET_CO_2_, end-tidal carbon dioxide tension; PaCO_2_-P_ET_CO_2_, arterial-to-end-tidal carbon dioxide tension gradient; P(A-a)O_2_, alveolar-to-arterial oxygen tension gradient; mmol, millimoles; nmol, nanomoles.

a median and (interquartile range); ^b^, *p*-value for Moods Median Test.

The thiafentanil-medetomidine combination required more frequent butorphanol rescue boluses (median: 2; interquartile range: 2–3) during the data collection period (time period between recumbency and data collection completion) compared to the etorphine-medetomidine (median: 0; interquartile range: 0–1) to rescue the impala from bouts of apnoea and/or respiratory distress (*p* = 0.001). Most butorphanol boluses (11 out of 23; *n* = 8 for thiafentanil-medetomidine and *n* = 3 for etorphine-medetomidine) were administered within 2 min after recumbency. Follow-up butorphanol boluses were only required in the thiafentanil-medetomidine combination and were administered at approximately 6 (7 out of 23) 10 (4 out of 23) and 15 (1 out of 23) min after recumbency. Despite the required rescue interventions, the respiratory rates (obtained in the boma and procedure room, between bouts of apnoea) and measured minute volumes (which were obtained after all apnoea events) were similar between the combinations (Table 1). It was noticed that the impalas that suffered apnoea were also much more rigid (hypertonic appendicular, thoracic and abdominal muscles) compared to those that did not suffer apnoea. Furthermore, the impalas appeared more relaxed soon after the butorphanol bolus. Even after successful apnoea treatment all impalas suffered from hypoxaemia, where mean PaO_2_ tensions were 47 mmHg (6.3 kPa) and 41 mmHg (5.5 kPa) for thiafentanil-medetomidine and etorphine-medetomidine, respectively (*p* = 0.193). Heart rates were higher when thiafentanil-medetomidine was used (*p* = 0.032) compared to etorphine-medetomidine, but the mean arterial blood pressures did not differ between the combinations (*p* = 0.226).

## Ethical considerations

The University of Pretoria’s Research and Animal Ethics Committee approved the study prior to commencement (V099/13 and V012/16). The wildlife and veterinary community will benefit from the results of this study by better understanding the physiological effects of using different potent opioid drug combinations during chemical capture. Fifteen healthy adult female impalas were purchased from an extensive game farm operation. They were captured and translocated to the boma for study. A 6-week pre-trial boma adaption period was observed. Experienced research and wildlife veterinarians conducted the study procedures. Upon completion of the trials, the impalas were recaptured and released onto an extensive game farm.

## Discussion

A number of important clinical findings need highlighting. The first novel finding is that the time to recumbency was no different when the thiafentanil-medetomidine combination was used compared to the etorphine-medetomidine combination. The second is that the more rigid the impala was (more so with thiafentanil-medetomidine) because of generalised muscle hypertonia, the more apnoea was noticed. This apnoea was successfully treated with one–three low doses of butorphanol. These doses did not cause arousal but improved breathing and reduced muscle rigidity. The third and final observation is that despite apparently normal temperature, heart rate and respiratory variables (rate and minute volume) once apnoea was resolved, all impalas suffered clinically significant hypoxaemia.

Thiafentanil has been advocated as the preferred potent opioid for immobilisation of free-ranging impalas because it causes rapid time to recumbency and fewer fatalities compared to when fentanyl and etorphine are used (Burroughs et al. 2012). Janssen et al. (1993) reported that thiafentanil (0.050 mg/kg) alone, at a similar dose to our study, induces immobilisation rapidly, with times to recumbency of 2.7 (± 0.6) min. Etorphine, when used alone, appears to demonstrate a dose-dependent decrease in times to recumbency, where a low dose (0.020 mg/kg) and high dose (0.070 mg/kg) induce recumbency in 12.9 (± 4.9) and 3.7 (± 1.4) min, respectively (Cheney & Hattingh 1987; Meyer et al. 2010). Furthermore, combinations of thiafentanil (0.032 mg/kg ± 0.0028 mg/kg)-medetomidine (0.053 mg/kg ± 0.0047 mg/kg) and etorphine (0.041 mg/kg ± 0.0035 mg/kg)-medetomidine (0.053 mg/kg ± 0.0047 mg/kg) cause recumbency in 4.9 (± 3.1) and 9.2 (± 3.8) min, respectively (Meyer et al. 2008). All current evidence indicates that thiafentanil alone, or in combination with sedatives or tranquillisers, is the potent opioid that achieves more rapid time to recumbency in impalas. However, in contrast we found that thiafentanil-medetomidine did not produce a more rapid time to recumbency compared to etorphine-medetomidine, despite using higher drug doses compared to the other studies. We assumed complete intramuscular injection of the drugs when dart placement was good and they fully discharged. However, we cannot rule out the possibility that the drugs could have been deposited into a fascial plane (between muscles and subcutis), which could explain poor drug absorption and therefore delayed onset of action. We used 3 mL volume darts, in order to keep the dart size consistent between all the protocols used in the entire drug trial study (Buck et al. 2017; Gerlach et al. 2017; Zeiler et al. 2015). One component of the entire drug trial study was to evaluate the effects of an etorphine-medetomidine-ketamine versus a butorphanol-medetomidine-ketamine immobilisation combination (reported elsewhere), which required up to 3.0 mL volume, therefore 3 mL darts were used during the entire drug trial study (Gerlach et al. 2017). However, the drug combinations reported in this study (total volume = 0.42 mL of drugs; 14% volume of the dart) were seven times diluted, with sterile water for injection, to increase the volume to 3 mL. The change in drug concentration could have altered the pharmacokinetics of the drugs mainly by reducing the rate of absorption from the intramuscular site, thus prolonging the time to recumbency, despite administering a total drug dose higher than those previously reported (Meyer et al. 2008).Unfortunately, *in vitro* and *in vivo* physicochemical interactions of the mix of the potent opioids, medetomidine and injectable sterile water were not determined or ruled out as possible causes of a slower clinical effect. However, similar mixtures, used at higher concentrations, did not appear to interact, or have reduced efficacy (Meyer et al. 2008). Our findings highlight that further studies are warranted to determine what the volume-concentration relationship effects are on speed of absorption and efficacy of potent opioids, and whether physicochemical interactions are present when mixing drugs in a dart.

Rapid times to recumbency are desirable when capturing impalas, or any other antelopes, because this is believed to translate into a decrease in capture-related morbidities, such as capture-induced hyperthermia and myopathies, which often develop into mortalities (Meyer et al. 2008). Etorphine (1.5 mg) and thiafentanil (1.2 mg) were used in combination with either azaperone (40 mg) or medetomidine (2 mg) (Meyer et al. 2008). The mean (±s.d.) time to recumbency and cortisol concentrations were 6.5 (± 3.6) min and 66.0 (± 48.6) nmol/L, respectively, averaged over the four drug combinations. Surprisingly, the higher dose of the potent opioids used in our study, compared to the Meyer et al. (2008) study, resulted in longer times to recumbency (13.1 ± 6.0 min) and higher cortisol concentrations (76.7 nmol/L ± 36.5 nmol/L), overall. Furthermore, in contrast to Meyer et al. (2008), in our study we found that the shorter the induction time the higher the cortisol concentration when the thiafentanil-medetomidine protocol was used, and did not find an association between these variables when the etorphine-medetomidine protocol was used. Meyer et al. (2008) made use of powder charged darts (Pneu-Darts) with barbed needles, which ensured rapid injection speeds and stability of the dart depth during injection. The darts (Dan-Inject darts, charged with compressed air, with plain needles) used in this study most likely injected the drugs more slowly and at a variable depth. The difference in the injection characteristic of the darts used could have also contributed to the prolonged induction times reported in our study. These contradictory findings highlight that further research is needed to determine the effects of a stress response on times to recumbency, but they also highlight the limitations of using a single serum cortisol concentration as an indicator of the magnitude of a stress response (Hart 2012).

The overall appendicular (King & Klingel 1965; Pienaar 1969; Seal et al. 1985), thoracic (Benthuysen et al. 1986; Haigh 1977; Pearce & Kock 1989; Weisner, Rietschel & Gatesman 1984) and abdominal rigidity is a frequently reported observation when potent opioids are used during chemical capture. Opioid-induced muscular rigidity remains a serious clinical challenge in many animals, including humans treated with potent opioids, especially when fentanyl and its analogues are used (Bowdle 1998). We observed that impalas that were more rigid tended to suffer more bouts of apnoea. Moreover, thiafentanil-medetomidine caused more profound rigidity compared to etorphine-medetomidine. The increased appendicular and abdominal muscle rigidity is thought to be mediated through the mu-opioid receptor type (Woolf et al. 1973). The rigidity of the respiratory muscles could be because of the potent opioids disrupting usual respiratory rhythm generation by interfering with the pre-Bötzinger complex (causing slow inspiratory effort or struggling to initiate an inspiratory effort) and Kölliker-Fuse nucleus (transition from inspiration to expiration) through the activation of mu-receptors (Pattinson 2008). The fact that thiafentanil specifically interacts with the mu-receptor (Yaksh & Wallace 2011) compared to etorphine could explain our observation that thiafentanil-medetomidine caused more profound muscle rigidity. Also, thiafentanil is likely to be more lipid soluble than etorphine (as fentanyl is compared to morphine) (Yaksh & Wallace 2011), which could have translated into higher central concentration of the drug interacting with the central mu-receptors in a shorter period of time, resulting in more profound effects on the respiratory system. Also, our study made use of equal total doses (2 mg) of thiafentanil and etorphine. It is claimed that thiafentanil has ‘twice the potency of etorphine’ (Lance & Kenny 2012), but there is no scientific evidence whether these drugs are equipotent or not. Therefore, it is plausible that the more profound muscle rigidity and respiratory depression in the thiafentanil-medetomidine group could have been as a result of administering this more potent opioid at the same dose of etorphine. The muscle rigidity caused by the opioids can be remedied by administering drugs with muscle relaxant properties (benzodiazepines and alpha_2_-adrenoceptor agonists) or drug with mu-antagonist properties (butorphanol). However, medetomidine did not appear to reduce this rigidity as would be expected from its prominent central muscle relaxant effects (Kästner 2006). Furthermore, butorphanol, a mixed mu-antagonist and kappa-agonist (at 1:1 potent opioid dose), tended to reduce muscle rigidity and resolved etorphine-induced apnoea more reliably than thiafentanil-induced apnoea. However, etorphine-induced apnoea (*n* = 3) was not as frequent in our study (*n* = 3 impalas) as thiafentanil-induced apnoea (*n* = 8). Also, multiple butorphanol doses were required in the thiafentanil-medetomidine immobilised impalas to completely resolve apnoea. Similar to findings reported in etorphine-immobilised goats (Haw, Meyer & Fuller 2016), butorphanol, at the doses used, did not induce arousal from immobilisation but caused muscle relaxation in the goats and improved their breathing rate and rhythm.

Overall, the impalas in our study demonstrated respiratory rates (between the bouts of apnoea) that were within normal limits (7–15 breaths per min) for antelopes immobilised with potent opioids (Harthoorn 1967). Also, our overall mean ± s.d. minute volumes were 8.5 L/min ± 4.0 L/min and were similar to etorphine-immobilised impalas (10.9 L/min; mean body weight 36 kg; Meyer et al. 2010) and those of small domestic ruminants (6.3 L/min – 10.4 L/min: Bakima et al. 1988; Hales & Webster 1967). Despite the adequate respiratory rates and minute volume, hypoxaemia and hypercapnia were observed. The marginally widened PaCO_2_-P_ET_CO_2_ (normal < 10 mmHg) and wide P(A-a)O_2_ (normal < 20 mmHg) gradients suggest that the origin of the hypoxaemia was more likely because of impedance of oxygen diffusion and physiological right-to-left intrapulmonary shunting of blood rather than dead-space ventilation. Etorphine, when administered to goats, caused pulmonary hypertension (mean pulmonary artery pressure 23 mmHg ± 6 mmHg), which is a plausible cause of oxygen diffusion deficits and right-to-left intrapulmonary shunting (Meyer et al. 2015; Vodoz et al. 2009). Alpha_2_-adrenoceptor agonists, in small stock, are also believed to cause pulmonary hypertension, or a rapid transit time of blood flow across the pulmonary capillary bed decreasing time for oxygen diffusion (Kästner 2006). Furthermore, medetomidine causes intense peripheral vasoconstriction mostly through its direct activation of alpha_2_-adrenoceptors in the endothelium. This vasoconstriction is thought to cause a delay in blood flow through peripheral tissues resulting in increased oxygen extraction (Kästner 2006). Thus, blood returning to the pulmonary capillary has a lower oxygen content, which then could translate into a wider than anticipated P(A-a)O_2_ gradient (Zeiler et al. 2015).

Thiafentanil, the latest potent opioid used for free-ranging ungulate capture, was introduced in the early 1990s (Janssen et al. 1993). Compared to etorphine, claims exist that thiafentanil immobilises animals more rapidly, is safer and causes less respiratory depression (Burroughs et al. 2012; Lance & Kenny 2012). However, our findings are contradictory, and therefore more investigation, using well-designed studies, are required to ensure a more complete understanding of the pharmacodynamic effects of thiafentanil and etorphine and their role in free-ranging ungulate capture.

## Limitations of the study

Unfortunately, serial samples of blood for serum cortisol and catecholamine (adrenalin and noradrenalin) concentration measurements were not obtained. This serial sampling would have allowed a more precise understanding of the magnitude of contribution that acute stress and increased sympathetic tone have on the overall cardiopulmonary effects of immobilisation and times to recumbency. The trials were not randomised as impalas were first immobilised with thiafentanil-medetomidine and then 4 weeks later with etorphine-medetomidine. Therefore, we cannot rule out the influence of the effects of time in the boma and data collection experience gained by the handlers on the results of the study. However, there were no statistical differences in the times when data were collected and the washout period was long enough to rule out residual drug effects (minimum washout period is 6 days). Therefore, we believe that this limitation had little to no influence on the outcomes of the study. Administering drugs by dart is prone to a number of errors, especially in flighty species such as impala. The operator can never be confident that all of the drugs were administered intramuscularly; therefore the limitations of darting should be recognised when interpreting data from any immobilisation study. In light of these limitations, it would have been invaluable to have hand-injected the impala and compare these findings to the data from darted impala (Smith et al. 1993). Furthermore, comparison of our findings to other studies was attempted in the discussion, but the reader should be aware of the limitations of comparing these studies because of differences in the animals themselves, population dynamics of the herd, conditions under which animals were immobilised, the degree of stress the animals experienced prior to dart placement, different dart types and rifles being used and operator’s experience in placing darts.

## Conclusion

The thiafentanil-medetomidine combination induced recumbency in times that were no different to those induced by etorphine-medetomidine, and both these times were longer than those seen in other studies and field captures. These unexpected prolonged times to recumbency were possibly because of the administration of diluted drugs (7 × dilution with sterile water for injection to fill a 3 mL dart) that caused a delay in intramuscular absorption because of a decreased absorption surface area to volume ratio. The effects of different stress responses may have also played a role. However, further research to clarify what primarily influences recumbency times is needed. The clinical implications of immobilising impalas using a potent opioid (especially thiafentanil) and medetomidine combination is that apnoea and profound muscle rigidity can be expected and that butorphanol (1:1 the potent opioid dose) is an effective rescue intervention that does not cause arousal. In addition, clinically relevant hypoxaemia and hypercapnia were present despite seemingly normal heart and respiratory rates and ventilation. The hypoxaemia and hypercapnia are suspected to be primarily because of pulmonary hypertension, causing gas diffusion deficits, and an increase in right-to-left intrapulmonary shunting of blood. Therefore, butorphanol and oxygen insufflation should be considered as essential rescue interventions for all impalas immobilised with these potent opioid combinations.
